# Supplement to the revision of the genus *Eremosaprinus* Ross, 1939 (Coleoptera, Histeridae, Saprininae): new distributional data and description of a new species from Arizona, U.S.A.

**DOI:** 10.3897/zookeys.409.4801

**Published:** 2014-05-14

**Authors:** Tomáš Lackner, Alexey K. Tishechkin

**Affiliations:** 1 Czech University of Life Sciences, Faculty of Forestry and Wood Sciences, Department of Forest Protection and Entomology, Kamýcká 1176, CZ-165 21 Praha 6 – Suchdol, Czech Republic; 2 Louisiana State Arthropod Museum, Department of Entomology, Louisiana State University, 404 Life Sciences Building, Baton Rouge, LA 70803-1710 USA

**Keywords:** *Eremosaprinus warneri*, new species, Coleoptera, Histeridae, Saprininae, North America, inquiliny

## Abstract

A new species of the genus *Eremosaprinus* Ross, 1939, *E. warneri*, is described from southeastern Arizona, USA, and incorporated into the identification key for the genus. Description of the new species is supplemented with SEM micrographs and drawings of sensory structures of the antenna and male genitalia. New distribution data on four species, *E. distinctus* Lundgren, 1992, *E. hubbardi* (Wenzel, 1939), *E. minimus* Tishechkin & Lackner, 2012, and *E. unguiculatus* (Ross, 1939), are also provided.

## Introduction

Shortly following the recently published revision of the obligate mammal burrow-dwelling genus *Eremosaprinus* Ross, 1939 (Tishechkin and Lackner 2012), study of more recently collected *Eremosaprinus* specimens from Arizona revealed an additional undescribed species of this genus. In this paper we describe the species, update the identification key for the genus and list the new distributional information on several other *Eremosaprinus* species. We refer readers to Tishechkin and Lackner (2012) for methods, conventions and abbreviations (LSAM is the abbreviation used here for the Louisiana State Arthropod Museum). References to the figures from that publication are made below in ***bold italic*.** All specimens of described species listed below were collected by W. B. Warner, unless specified otherwise, and are deposited in CWBW.

### Revised key to *Eremosaprinus* species (sensu Tishechkin and Lackner 2012)

The original key needs to be edited beginning with couplet 6. After studying a long series of the new species and revisiting the characters of *Eremosaprinus distinctus* Lundgren, 1992, *Eremosaprinus minutus* Tishechkin & Lackner, 2012, *Eremosaprinus rossi* Tishechkin & Lackner, 2012 and *Eremosaprinus verityi* Tishechkin & Lackner, 2012, we decided to drop from this couplet the mention of the dorsal microsculpture characters. The new species is very similar to *Eremosaprinus rossi* in overall appearance and has distinct background microsculpture, while in the only known *Eremosaprinus rossi* specimen it is almost nonexistent. We suspect, however, that the discovery of more *Eremosaprinus rossi* specimens might prove this species also possesses relatively distinct background microsculpture (as the new species does), the holotype appearing to be a somewhat worn specimen. So, to avoid possible confusion, we delete the mention of microsculpture characters from the key and use only unambiguous male characters. However, diagnosis of the so far unknown females of *Eremosaprinus rossi* might prove to be problematic without a reliable male association.

**Table:** 

6	Males with pair of short, obtuse, triangular tubercles on posterior margin of metaventrite (***fig. 42***); protibia with at least three teeth on outer margins	7
–	Males without paired tubercles on posterior margin of metaventrite (***fig. 112***), but with two tubercles on basal end of abdominal setose patch (***fig. 119***); carinal prosternal striae widely separated, strongly concave; protibia with only two large teeth on outer margins	*Eremosaprinus rossi* Tishechkin & Lackner, 2012
7	Fourth dorsal elytral striae anteriorly not connected with abbreviated sutural elytral striae; impression on metaventrites of males deep, circular; disc of 1^st^ abdominal ventrite in males without a setose patch; carinal prosternal striae anteriorly united in semicircular loop (***fig. 41***)	*Eremosaprinus distinctus* Lundgren, 1992
–	Fourth dorsal elytral striae anteriorly connected with complete sutural elytral striae under smooth arch; impression on metaventrites in males shallow, elongate or short triangular; disc of 1^st^ abdominal ventrite in males medially with a setose patch (***fig. 155***); carinal prosternal striae not united anteriorly	8
8	Impressions of metaventrite and 1^st^ abdominal ventrite of males relatively small, occupying about half the lengths of metaventrite and 1^st^ abdominal ventrite (occasionally up to two-thirds the length of 1^st^ abdominal ventrite) (***figs 92, 149***); dorsal punctures small and sparse, especially on anterior part of elytral disc	9
–	Males with wide and long setose impressions occupying entire length of metaventrite and 1^st^ abdominal ventrite (Fig. 4); dorsal punctures large and dense throughout the surface (Fig. 1)	*Eremosaprinus warneri* sp. n.
9	Larger species, PEL: 2.70–3.60 mm; body dorsally entirely black; punctation of pygidium sparser, interspaces between punctures larger than punctures themselves; opaque microsculpture of pronotal and elytral discs sparse, inconspicuous; male genitalia (***figs 157–165***) compare with those in ***figs 100–108***; known exclusively from California (Inyo and San Bernardino Counties)	*Eremosaprinus verityi* Tishechkin & Lackner, 2012
–	Smaller species, PEL: 2.10–2.70 mm; body, especially pronotum dorsally paler, brownish; punctation of pygidium denser, punctures separated by approximately their diameter; opaque microsculpture of pronotal and elytral discs dense and conspicuous; male genitalia as on ***figs 100–108***; known exclusively from Arizona (Cochise, Mohave, La Paz, Pima and Yuma Counties)	*Eremosaprinus minimus* Tishechkin & Lackner, 2012

## Taxonomy

### Eremosaprinus warneri sp. n.

http://zoobank.org/163E10DA-BBDF-4423-95FC-5C30F1B67D7F

http://species-id.net/wiki/Eremosaprinus_warneri

**Figures 1–5.**
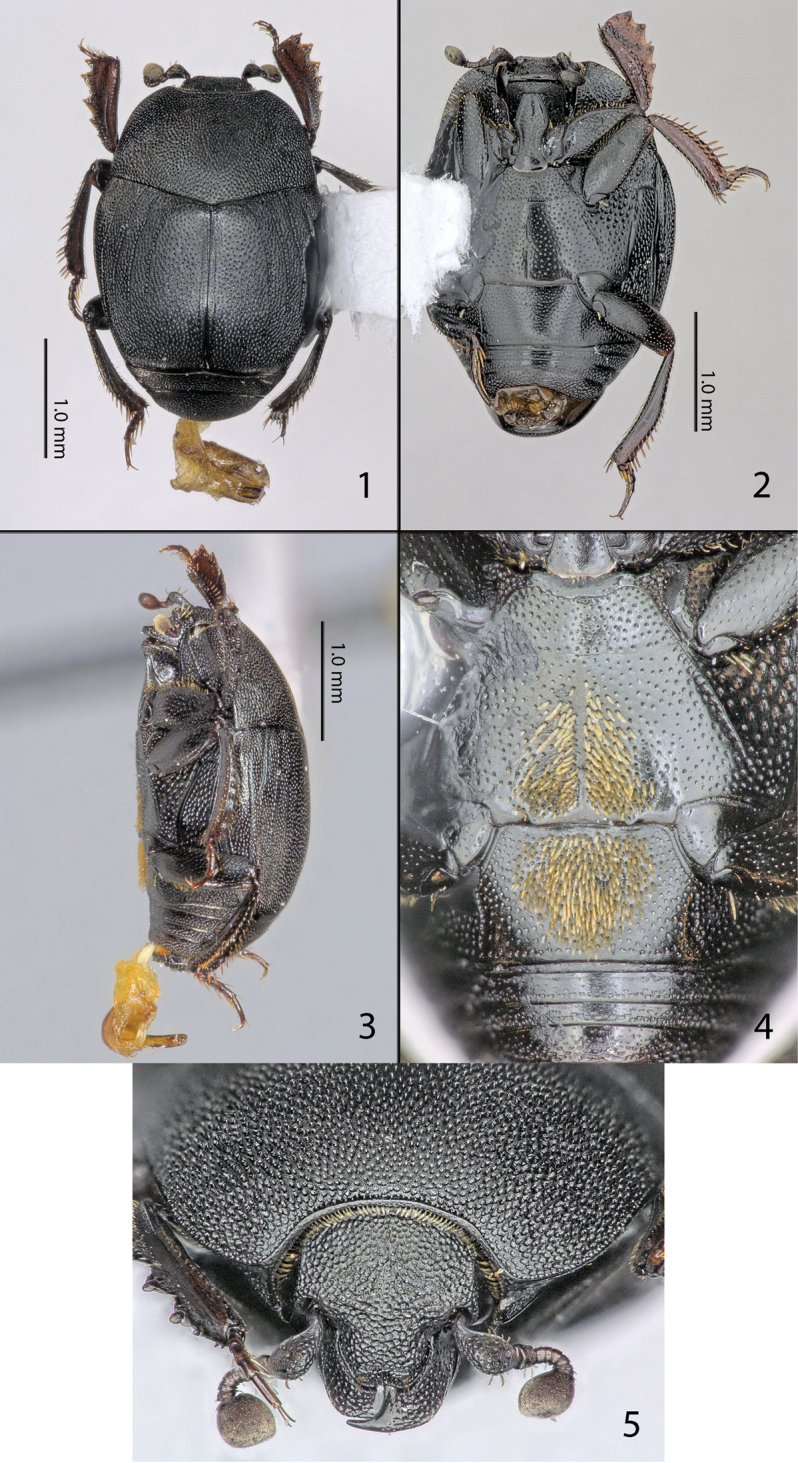
**1** *Eremosaprinus warneri* sp. n., habitus, dorsal view. **2** ditto, ventral view **3** ditto, lateral view **4** *Eremosaprinus warneri* sp. n., male, metaventrite + first abdominal ventrite **5** *Eremosaprinus warneri* sp. n., head, frontal view.

Figs 1 [Fig F2] [Fig F3] –23

#### Type material.

**Holotype**, point-mounted male, labeled “USA: AZ: Cochise Co., Bagby Rd., 0.2 mi W Central Hwy.; 31°33'14"N, 109°42'06"W; viii.28.–x.9.2011; barrier pitfalls; W. B. Warner / HOLOTYPE *Eremosaprinus warneri* sp. n. A.K.Tishechkin & T.Lackner des. 2012” (FMNH). **Paratypes** (46, in CTLA, CWBW, FMNH and LSAM): 6 ♂♂ and 12 ♀♀ females labeled as the holotype; 3 ♂♂ and 1 ♀ as preceding, but collected on 28.viii.–19.xi.2011; 4 ♀♀ with the same locality, collector and collecting method, but collected on 9.x.–19.xi.2011; 2 ♀♀ with the same locality, collector and collecting method, but collected on 12.viii.–5.ix.2012; 3 ♂♂ and 6 ♀♀ with the same locality, collector and collecting method, but collected on 5.ix.–6.x.2012; 3 ♀♀ from Arizona, Cochise Co., Birch Rd., 4.1 mi E of Highway 191 at 31°58'43"N, 109°46'41"W, collected by black cup pitfall traps on 28.vii.–9.x.2011 by W. B. Warner; 1 ♂ and 4 ♀♀ from Arizona, Cochise Co., 1.5 mi S junction of Highways 191 and 181 at 31°51'44"N, 109°41'59"W, collected by black cup barrier pitfall traps on 28.viii.–9.x.2011 by W. B. Warner; 2 ♂♂ and 1 ♀ with the same locality, collector and collecting method, but collected on 9.x.–19.xi.2011.

#### Diagnosis.

In general appearance, *Eremosaprinus warneri* has a close resemblance to the apparently sympatric *Eremosaprinus rossi*, both species possessing similar elytral strial patterns, dense dorsal punctation and a large elongate setose impression on the metaventrite and 1^st^ abdominal ventrite in males. Furthermore, the two species share a synapomorphy of a microscopic slit-like aperture situated near the anterior margin of prosternum, just before the deep transverse sulcus across prosternal keel; this aperture can possibly be interpreted as a homology to the tiny foveae found in other congeners, which these species otherwise lack. The new species can be diagnosed by the presence of a pair of tubercles on the posterior margin of the male metaventrite in *Eremosaprinus warneri* (absent in *Eremosaprinus rossi*), the presence of a pair of tubercles on the posterior margin of the male 1^st^ abdominal ventrite in *Eremosaprinus rossi* (absent in *Eremosaprinus warneri*) and the shape of the prosternal carinal striae (forming an almost circular loop in *Eremosaprinus rossi*, with a more elongate and narrow loop in *Eremosaprinus warneri*, compare Figs 9 and ***112***).

#### Description.

Body measurements: PEL: 2.4–3.3 mm; APW: 0.9–1.2 mm; PPW: 1.7–2.3 mm; EL: 1.5–2.1 mm; EW: 2.0–2.7 mm. Body (Figs 1–3) wide oval, convex, ventral surface flattened. Color dark brownish black, legs and antennae paler, antennal clubs dark rufous.

**Figure 6. F2:**
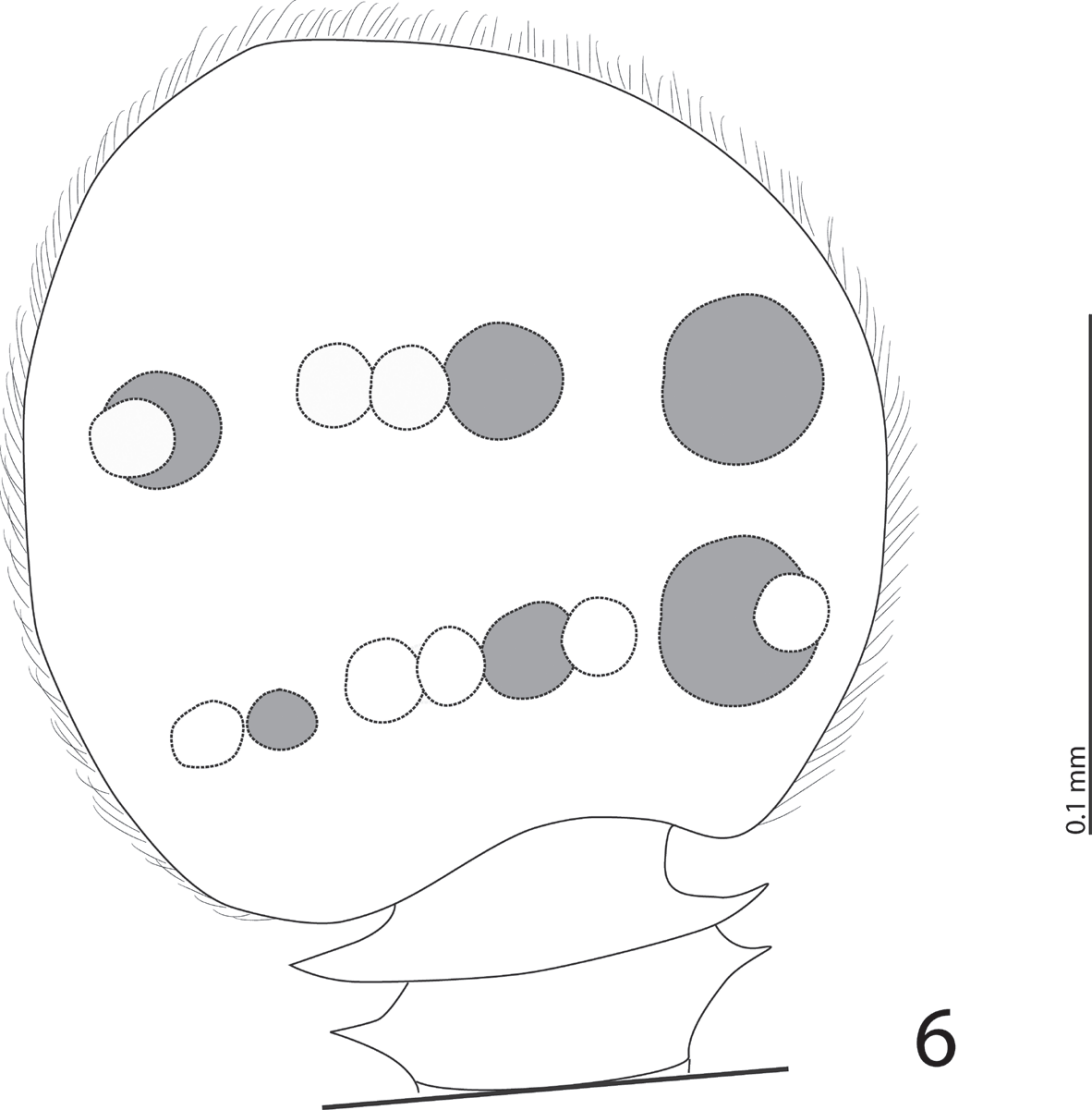
*Eremosaprinus warneri* sp. n., left antennal club, dorsal view (white circles show sensory areas and corresponding vesicles of dorsal side, grey circles depict sensory areas and vesicles of ventral side).

**Figures 7–14. F3:**
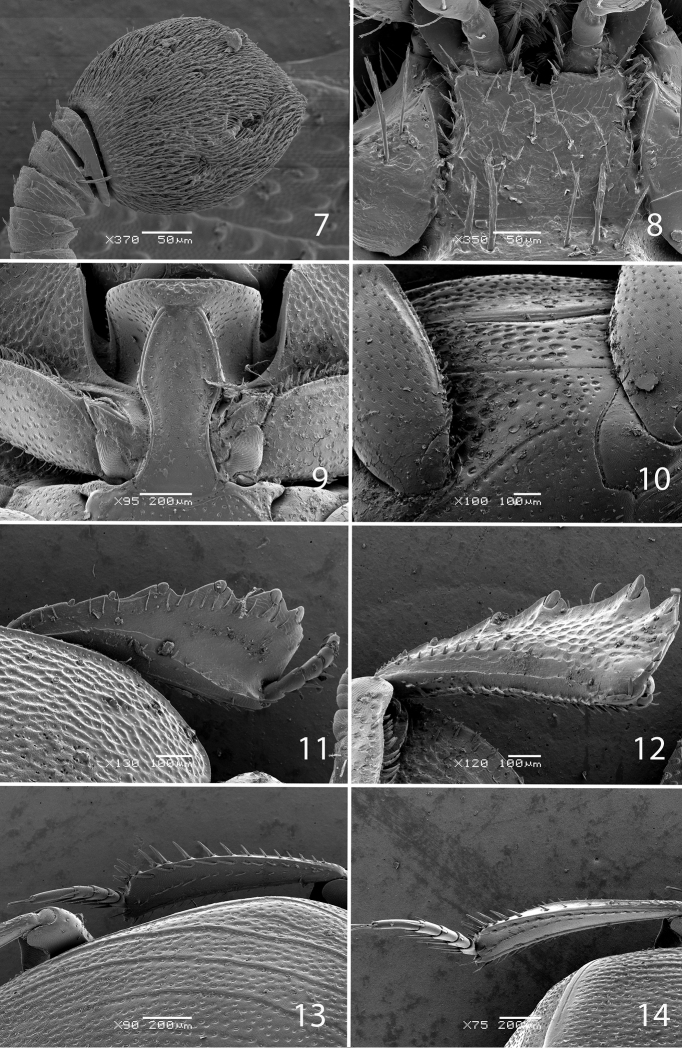
**7** *Eremosaprinus warneri* sp. n., left antennal club, ventral view. **8** *Eremosaprinus warneri* sp. n., mentum, ventral view **9** *Eremosaprinus warneri* sp. n., prosternum **10** *Eremosaprinus warneri* sp. n., lateral disk of metaventrite + metepisternum **11** *Eremosaprinus warneri* sp. n., protibia, dorsal view **12** ditto, ventral view **13** *Eremosaprinus warneri* sp. n., mesotibia, dorsal view **14** *Eremosaprinus warneri* sp. n., metatibia, dorsal view.

Antennae short, scape subquadrate, subequal to funicle in length; dorsal side with multiple long setae; pedicel enlarged, subquadrate. Antennal club (Fig. 7) small, spherical, slightly pointed apically, completely covered with short appressed tomentose sensilla intermingled with sparse short sub-erect sensilla. Antennal clubs ventrally with two rows of circular sensory areas situated above each other with three areas in each row; areas increased in size mediad; sensory areas dorsally likewise arranged in two rows, but areas of both dorsal rows approximately identical in size, circular; lower row contains five sensory areas while upper row contains three; sensory structures of antennal club (Fig. 6) in form of vesicles corresponding in number and shape to the sensory areas on the surface; no main vesicle “*v*” apparent.

Mouthparts: mandibles (Fig. 5) regularly rounded, with finely microsculptured surface and dense shallow punctures; mandibular apex acute and long, sub-apical tooth on left mandible small and obtuse. Labrum short, triangular, surface finely granulate; mentum (Fig. 8) subquadrate, anterior angles slightly produced, anterior margin medially with a tiny notch, surface around it with several setae; disc of mentum imbricate; lateral margins with a row of sparse ramose setae.

Frons (Fig. 5) moderately convex, with dense small anastomosing rugose punctures. Frontal stria absent; occipital stria complete, thin, carinate; supraorbital stria present along dorsal halves of inner margins of eyes, inconspicuous among rugose frontal punctation. Clypeus convex, lateral sides weakly so, anterior margin shallowly concave, its punctation slightly smaller that of frons.

Pronotum (Fig. 1) widest at base, lateral margins almost straight, anterior angles obtuse, narrowly rounded. Marginal pronotal stria poorly distinct among dense punctation laterally, completely absent behind head. Anterior margin of pronotum with a fringe of tiny thin setae. Pronotal disc singly microsculptured, with moderate to large shallow dense punctures, interspaces between them smaller than punctures themselves, progressively becoming denser, somewhat larger and more elongate laterally, turning into fine longitudinal wrinkles in lateral thirds of pronotal width. Ante-scutellar depression indistinct; scutellum minute, triangular; pronotal hypomeron with background microsculpture and dense shallow longitudinally arranged punctures, with numerous short yellow setae.

Elytra (Fig. 1) with fine background micro-sculpture, completely covered with small to medium shallow, mostly elongate dense punctures, their interspaces approximately half as large as punctures themselves, but can be larger; being progressively larger and denser posteriorly and laterally, with a tendency to merge into shallow longitudinal wrinkles in elytral intervals and on posterior fourth. Elytral striae thin, finely punctuate, weakly costate. Outer subhumeral striae absent, humeral striae indistinct, mostly obscured by dense punctation, inner subhumeral striae complete, running from bases to near apices, but not attaining apex. First dorsal elytral striae almost complete, 2^nd^ to 4^th^ long, entering posterior fifth, sub-equal in lengths, 4^th^ dorsal elytral stria being usually somewhat shorter. No traces of 5^th^ dorsal elytral striae present, sutural elytral striae complete, connected with 4^th^ dorsal elytral striae under angulated wide arch, occasionally very narrowly interrupted anteriorly. Apical elytral striae absent; marginal elytral striae thin, slightly carinate, complete; marginal epipleural striae thin, distinct and complete. Elytral epipleura with punctation identical to lateral areas of elytra in dorsal parts, areas between marginal elytral and epipleural striae smooth and shiny, with few small punctures.

Propygidium narrow, with small shallow circular anostomosing punctures; pygidium long and moderately convex, with elongate anostomosing punctures similar to ones on posterior parts of elytra, progressively denser and wrinkle-like apically.

Anterior margin of median portion of prosternum (Fig. 9) straight, not elevated, marginal prosternal stria indistinct. Prosternal process laterally with dense small punctures in anterior third, dorsally with tiny scattered punctures, slightly convex. Carinal prosternal striae long, convergent between procoxae, thence moderately convex, approaching each other anteriorly, terminating freely next to microscopic slit-like aperture situated near anterior margin, just before deep transverse sulcus across prosternal keel (Fig. 9). Lateral prosternal striae absent. Carinal profile shallowly concave, with weak notch at transverse anterior sulcus.

Mesoventrite transverse, with small, shallow, moderately dense punctures throughout disc, anterior margin deeply concave. Marginal mesoventral stria thin, complete. Meso-metaventral sutural stria absent; meso-metaventral suture thin, distinct. Metaventrite long, with slightly larger punctures than those of mesoventrite. Metaventral profile weakly concave in males, slightly convex in females. Longitudinal suture of metaventrite distinct, complete, thin and impunctate. Most of discs of metaventrite and 1^st^ abdominal sternite in males occupied by relatively deep elongate oval setose patch (Fig. 4). Setae of this patch flattened and dorso-ventrally appressed, cuticle mostly carpeted by setal cover; metaventral part with narrow weak keel along longitudinal suture. Lateral striae of metaventrite long, slightly abbreviated before metacoxa, punctuate, postmesocoxal striae represented by short outer fragment near anterior end of metepisternum. Lateral discs of metaventrite (Fig. 10) with large shallow dense (0.3–1.2), circular punctures; metepisternal discs with smaller denser punctures, becoming progressively sparser posteriorly. First abdominal ventrite of males mostly occupied by setose depression (Fig. 4), in females this setose depression absent, completely striate laterally; peripheral areas outside of depression with identical type of punctation as peripheral parts of disc of metaventrite.

All femora smooth and sparsely punctuate, wide; punctures of profemora slightly larger and denser than those of meso- and metafemora; profemora with a row of short stiff setae on both margins. Margins of meso- and metafemora with rows of shorter setae on dorsal sides, hidden from ventral aspect, an extra row of short stiff setae present on posterior margins.

Protibia (Fig. 11) rather wide, outer margins with four shallow teeth in anterior halves topped with small flattened denticles and with extra one or two tiny denticles toward bases. Anterior margins with one tiny denticle. Anterior surface of protibia with complete anterior protibial stria and with two complete rows of rather long sparse setae. Protarsal groove deep; protibial spur tiny, inconspicuous. Posterior surfaces of protibia (Fig. 12) with outer portion irregularly rugosely punctate, clearly distinct from smooth narrow median portion, separated by irregular carinate elevation with a row of microscopic setae; posterior protibial stria complete, thin and carinate, on anterior half with a row of microscopic setae; setae of inner row double: one row of setae short and stiff; another row of setae approximately identical in length, but setae much thinner.

Mesotibia (Fig. 13) long and narrow, distinctly curved, outer margin with a row of tiny denticles and a row of long denticles, progressively increasing in size distally; setae of outer and median rows long and sparsely spaced. Anterior surfaces smooth, mesotibial spurs tiny and inconspicuous. Anterior and posterior mesotibial striae complete. Tibial apices with several relatively long thin denticles. Metatibia (Fig. 14) in all aspects similar to mesotibia, but slightly longer and thinner. All tarsi long and thin, meso- and metatarsomeres each with long (1.4–1.7 times corresponding tarsomere length) straight bristles ventrally and shorter, thinner bristle dorsally. First meso- and metatarsomeres with three ventral bristles, 2^nd^ to 4^th^ with a pair of such bristles, being uneven in length (outer ones distinctly shorter). Tarsal claws long, nearly straight, 0.5 times length of correspondent apical tarsomere.

**Figures 15–23.**
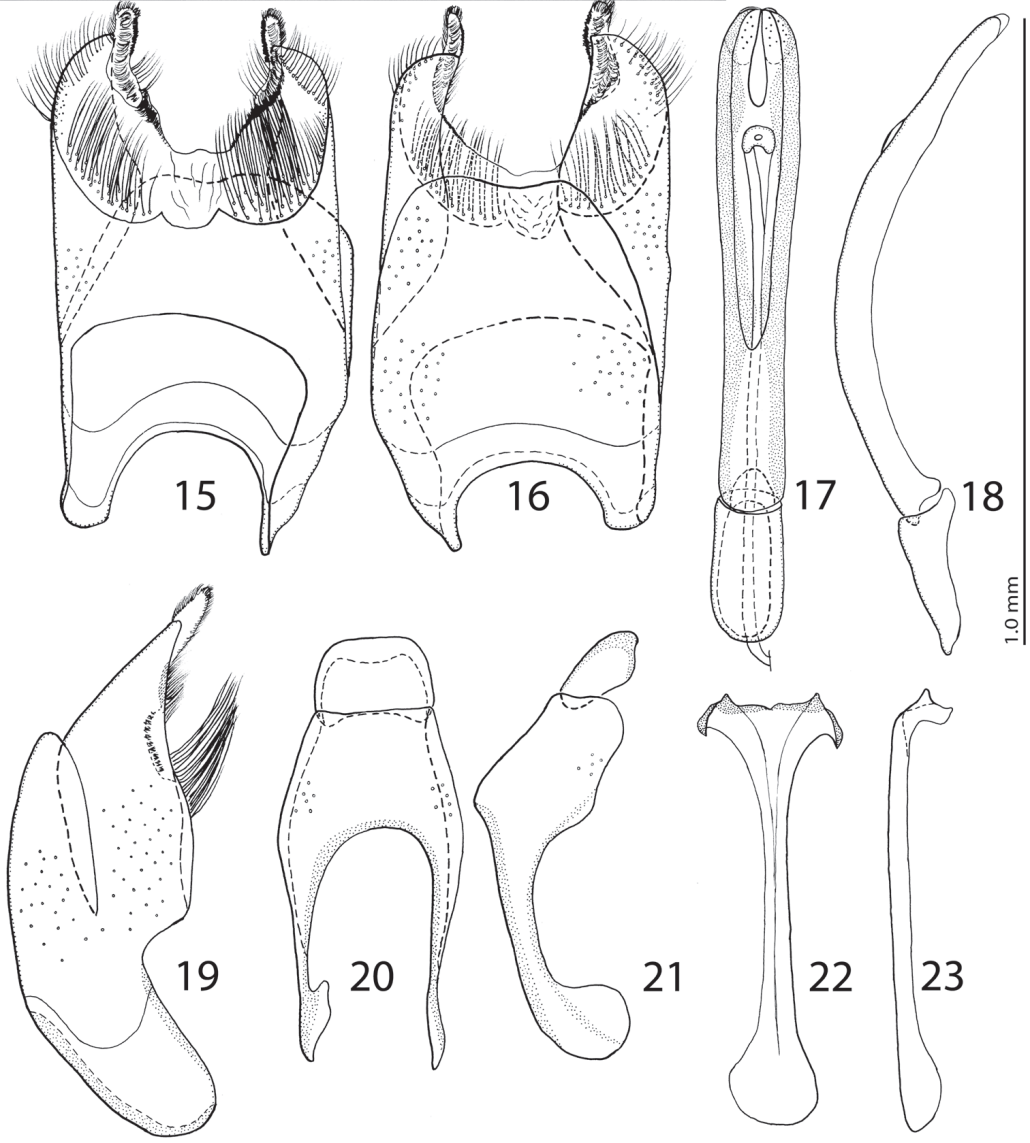
**15** *Eremosaprinus warneri* sp. n., 8^th^ sternite and tergite, ventral view. **16** ditto, dorsal view **17** *Eremosaprinus warneri* sp. n., aedeagus, dorsal view **18** ditto, lateral view **19** *Eremosaprinus warneri* sp. n., 8^th^ sternite and tergite, lateral view **20** *Eremosaprinus warneri* sp. n., 9^th^ + 10^th^ tergites, dorsal view **21** ditto, lateral view **22** *Eremosaprinus warneri* sp. n., spiculum gastrale, ventral view **23** ditto, lateral view.

Male genitalia: Eighth sternite entirely fused medially (Figs 15–16), medio-laterally with few pores, apically with rather large vela basally with two brushes of long, dense setae (most easily visible from lateral view, Fig. 19); two rows of shorter setae appear apically. Apices of 8^th^ sternite with two small elongate membranous patches covered with microscopic setae. Apical part of 8^th^ tergite medially faintly inwardly arcuate (Fig. 16); 8^th^ tergite medio-laterally with pores. Eighth sternite and tergite fused laterally (Fig. 19). Ninth tergite (Fig. 20) laterally with few pores, apical margin almost straight, with a conspicuous median projection observable from ventral view (Fig. 21); 10^th^ tergite basally inwardly arcuate. Spiculum gastrale (Figs 22–23) almost parallel-sided with dilated ends; apical end strongly sclerotized, with two horn-like projections; basal end of spiculum gastrale outwardly arcuate. Aedeagus (Figs 17–18) strongly sclerotized, almost parallel-sided on basal half, slightly thickened on apical half; convergent apically. Parameres fused on approximately basal third, aedeagus strongly curved ventrad (Fig. 18). Basal piece of aedeagus short, its ratio to parameres length approximately 1:4.

#### Remarks.

Sensory structures of the antenna of *Eremosaprinus warneri* differ substantially from those of the type species of the genus, *Eremosaprinus unguiculatus* (Ross, 1939); compare Fig. 6 with that of Tishechkin and Lackner (2012: ***fig. 138***). Such disparity between the species of *Eremosaprinus* was discussed previously (Tishechkin and Lackner 2012: 50) and the unity of the genus remains unclear. We prefer to keep this newly described species tentatively in the genus pending larger series of all already known species – something that would enable us to study sensory organs of the antennae among all species and allow us to reconstruct the phylogeny of the genus on species level.

#### Etymology.

We dedicate this species to our colleague and friend Bill Warner, an enthusiastic student and collector of Arizona beetles, histerids in particular, who collected the entire type series of this species and many other *Eremosaprinus* specimens.

#### Biology.

According to the collector, W.B. Warner, the type series of this species was collected using barrier and black pitfall traps that were set around banner-tail kangaroo rat (*Dipodomys spectabilis* Merriam, 1890) burrows (mostly within 100 cm radius of the burrow entrances). The new species is most likely an inquiline inhabiting the burrows of the above-mentioned rodent (W.B. Warner, pers. comm).

#### Distribution.

USA, Arizona.

### New data on the distribution of *Eremosaprinus* species

#### Eremosaprinus hubbardi

(Wenzel, 1939)

##### Material studied.

1 specimen: Arizona, La Paz Co., dunes 11 mi S Ehrenberg, 33°28'03"N, 114°36'26"W, 12.ii.-4.iii.2012; 6 specimens: Arizona, Maricopa Co., nr. Agua Caliente, 32°57'00"N, 113°17'31"W, 11.ii.–25.iii.2012; 11 specimens: Arizona, Yuma Co., SW of Dateland, 32°47'25"N, 113°32'54"W, 11.ii.–25.iii.2012; 1 specimen: Arizona, Yuma Co., 6 mi. N Gila Rd. on Hwy. 95, 32°50'23"N, 114°22'07"W, 12.ii.-4.iii.2012; 2 specimens: California, Riverside Co., Wiley Well Rest Stop on I-10, 33°36'18"N, 114°54'15"W, 18.ii.–4.iii.2012; 14 specimens: California, San Bernardino Co., Rt. 62, 12 mi ENE junction with Rt. 177, J. Saulnier, 30.xi.–22.xii.2011. First records for La Paz and Maricopa Counties, Arizona.

#### Eremosaprinus distinctus

Lundgren, 1992

##### Material studied.

1 specimen: California, San Bernardino Co., Rt. 62, 12 mi ENE junction with Rt. 177, J. Saulnier, 30.xi.-22.xii.2011. First record from San Bernardino Co., California.

#### Eremosaprinus minimus

Tishechkin & Lackner, 2012

##### Material studied.

40 specimens: Arizona, Cochise Co., Bagby Rd., 0.2 mi W Central Hwy., 31°33'14"N, 109°42'06"W, 28.viii.2011-10.vi.2012; 31 specimens: Arizona, Cochise Co., Birch Rd., 4.1 mi E Hwy. 191, 31°58'43"N, 109°46'41"W, 28.viii.2011-10.vi.2012; 2 specimens: Arizona, Cochise Co., 1.5mi.S jct.Hwys.191 and 181, 31°51'44"N, 109°41'59"W, 9.x.-19.xi.2011; 1 specimens: Arizona, Cochise Co., 3 mi.S Wilcox, C. W. O’Brien, 16.i.2008; 1 specimen: Arizona, La Paz Co., Ave. 51E, 0.5mi S of I-10, 33°37'27"N, 113°45'58"W, 12.ii.-4.iii.2012; 2 specimens: Arizona, Yuma Co., SW of Dateland, 32°47'25"N, 113°32'54"W, 11.ii.-25.iii.2012. First record for La Paz Co., Arizona.

#### Eremosaprinus unguiculatus

(Ross, 1939)

##### Material studied.

7 specimens: Arizona, La Paz Co., dunes 11 mi S Ehrenberg, 33°28'03"N, 114°36'26"W, 12.ii.–4.iii.2012; 57 specimens: Arizona, Maricopa Co., nr. Agua Caliente, 32°57'00"N, 113°17'31"W, 11.ii.–25.iii.2012; 1 specimen: California, Riverside Co., Wiley Well Rest Stop on I-10, 33°36'18"N, 114°54'15"W, 18.ii.-4.iii.2012. First records for La Paz and Maricopa Co., Arizona.

## Supplementary Material

XML Treatment for Eremosaprinus warneri

XML Treatment for Eremosaprinus hubbardi

XML Treatment for Eremosaprinus distinctus

XML Treatment for Eremosaprinus minimus

XML Treatment for Eremosaprinus unguiculatus
